# The role of lymphocyte-monocyte ratio on axial spondyloarthritis diagnosis and sacroiliitis staging

**DOI:** 10.1186/s12891-021-03973-8

**Published:** 2021-01-16

**Authors:** Jing Wang, Jinyu Su, Yuan Yuan, Xiaxia Jin, Bo Shen, Guoguang Lu

**Affiliations:** 1 Department of Clinical Laboratory, Taizhou Hospital of Zhejiang Province affiliated to Wenzhou Medical University, 150 Ximen Road, Linhai, Taizhou, Zhejiang Province China; 2 Department of Pharmacy, Taizhou Hospital of Zhejiang Province affiliated to Wenzhou Medical University, 150 Ximen Road, Linhai, Taizhou, Zhejiang Province China

**Keywords:** Axial spondyloarthritis (axial SpA), Lymphocyte-monocyte ratio (LMR), X-ray, Disease activity, Stage

## Abstract

**Background:**

Axial spondyloarthritis (axial SpA) is a chronic inflammatory disorder could lead to disability due to the failure of timely treatment. The role of lymphocyte-to-monocyte ratio (LMR) in axial SpA remains unclear. The aim of this study was to investigate the role of LMR in axial SpA diagnosis, disease activity classification and sacroiliitis staging.

**Methods:**

Seventy-eight axial SpA patients [51males and 27 females; mean age 41.0 (29–52) years] and 78 healthy controls (HCs) [55males and 23 females; mean age 40 (30–53) years] were enrolled in this study. The diagnosis of axial SpA was performed according to the New York criteria or the Assessment of Spondyloarthritis international Society (ASAS) classification criteria, whereas the staging of sacroiliitis in axial SpA patients was determined by X-ray examination. Comparisons of LMR levels between groups were performed using t test. Pearson or Spearman correlation analysis were used to assess correlations between LMR and other indicators. Receiver operating characteristic (ROC) curves were used to determine the role of LMR in the diagnosis of axial SpA.

**Results:**

Higher neutrophil-to-lymphocyte ratio(NLR), red blood cell distribution width(RDW), platelet-to-lymphocyte ratio(PLR), mean platelet volume(MPV), erythrocyte sedimentation rate (ESR), and C-reactive protein(CRP) levels and lower red blood cell (RBC), hemoglobin (Hb), Hematocrit (Hct), LMR, alanine aminotransferase (ALT), aspartate aminotransferase (AST), total bilirubin (TBIL) and albumin/globulin (A/G) levels were noted in axial SpA patients compared to HCs. Positive correlations were observed between LMR and RBC, Hb, Hct and A/G, whereas negative correlations were found between LMR and NLR, PLR, AST, and TBIL (*P < 0.05*). ROC curves showed that the area under the curve (AUC) for LMR in the diagnosis of ankylosing spondylitis was 0.803 *(95% CI = 0.734–0.872)* with a sensitivity and specificity of 62.8 and 87.2%, respectively, and the AUC (95% CI) for the combination of ESR, CRP and LMR was 0.975 (0.948–1.000) with a sensitivity and specificity of 94.9 and 97.4%, respectively. LMR levels were lower (*P < 0.05*) and significant differences in LMR values were observed among different stages (P < 0.05).

**Conclusions:**

Our study suggested that LMR might be an important inflammatory marker to identify axial SpA and assess disease activity and X-ray stage of sacroiliitis.

## Background

Axial spondyloarthritis (axial SpA), an immune-mediated chronic inflammatory rheumatic disease with unknown etiology, mainly affects the axial bone and articular structures, but also enthesitis, arthritis and dactylitis [1]. The prevalence of axial SpA is approximately 0.2–0.3%, and this condition primarily occurs in males aged 20–30 years. Without effective treatment, severe disabilities could develop in approximately one-third of patients [2]. To date, the pathophysiology of axial SpA is not completely understood. Risk factors associated with heredity factors, immunity and inflammation are considered the most important factors in the pathogenesis of axial SpA. The Assessment in Spondyloarthritis International Society (ASAS) provides recommendations for the management of axial SpA, including classification criteria, magnetic resonance imaging (MRI), X-rays and laboratory indicators for sacroiliac joints and the spine.

In current clinical practice, HLA-B27 is helpful in diagnosis of axial SpA given its high prevalence (90–95%) in axial SpA and its direct role in the onset of axial SpA [3]. In addition to HLA-B27, imaging modalities such as X-ray, computed tomography (CT), ultrasonography and MRI are typically employed in the diagnosis of axial SpA [4]. X-ray imaging is used to diagnose typical sacroiliitis, but early sacroiliitis is easily missed by this methodology. CT can satisfactorily reveal the sacroiliac joint space and articular surface bone, and reveal slight articular surface bone erosion and subchondral cystic change that X-rays cannot show. Ultrasonography can show peripheral arthritis and enthesitis, but it cannot evaluate axial manifestations. MRI can directly display articular cartilage, and is superior to CT in the early detection of sacroiliac joint cartilage changes, evaluation of sacroiliitis conditions and curative effect judgments [5]. Different imaging modalities have different characteristics and unique advantages and limitations, including radioactive properties, relative costs, long turnaround time and limited use in specific patients (such as pregnant women). Therefore, specific and sensitive biochemical markers for auxiliary diagnosis, treatment guidance and prognosis monitoring of axial SpA are urgently needed.

Bath ankylosing spondylitis disease activity index (BASDAI) and ankylosing spondylitis disease activity score (ASDAS) are common disease activity scoring systems for axial SpA [6]. Among them, BASDAI is based on the patient’s own feelings and do not include the doctor’s evaluation of the patient’s condition and laboratory indicators. ASDAS reflects the subjective feelings of patients and doctors, and includes inflammatory indicators, so this metric is more suitable for clinical practice. However, due to other complications, the increase in inflammatory markers can affect the evaluation of disease activity. Commonly used inflammatory markers, including erythrocyte sedimentation rate(ESR) and C-reactive protein(CRP) [7], have been verified to be related to axial SpA disease severity. ESR × duration of disease and CRP × duration of disease exhibit a good correlation with poor physical activity of axial SpA patients [8]. In recent years, some new inflammatory markers, such as the neutrophil to lymphocyte ratio (NLR) and red blood cell distribution width (RDW), have also been found to be associated with axial SpA disease activity. In axial SpA patients, NLR exhibits a good correlation with ESR and CRP, and increased NLR was found in patients with high disease activity [9]. In addition, different NLR levels were found in patients subject to different treatments, such as anti-TNF-alpha therapy, and nonsteroidal anti-inflammatory drugs [10]. Moreover, a significant difference in RDW was noted between patients with BASDAI index > 4 and < 4. RDW was positively correlated with BASDAI index as well as ESR and CRP levels [9]. Based on these findings, routine blood test indexes could represent potential resource for novel and effective marker exploration for axial SpA.

Like RDW and NLR, the Lymphocyte to monocyte ratio (LMR), is also a common blood routine indicator. LMR has been a subject of great interest in a wide range of fields such as inflammation, immunology and carcinoma for a long period of time. Recent data from several studies suggested that LMR was associated with diagnostic, pretreatment and prognostic statue of diseases. A genome-wide association study has confirmed that mutations in ITGA4 and HLA-DRB1 genes could affect LMR levels, and these genes have been widely recognized as susceptibility genes for autoimmune diseases, such as rheumatoid arthritis (RA) [11], suggesting their potential value in axial SpA diagnosis and prognostic evaluation.

To date, few studies have investigated the association between LMR and axial SpA. Therefore, the aim of this study was to explore the diagnostic value of LMR in axial SpA and its role in reflecting disease activity and X-ray staging.

## Methods

### Patients with axial SpA

A total of 78 patients with axial SpA [51males and 27 females; mean age 41.0 (29–52) years] were enrolled in this cross-sectional study. These patients received treatment at the Department of Endocrinology and Rheumatology, Taizhou Hospital (Zhejiang, China). All patients fulfilled the axial SpA criteria prescribed by the New York criteria of 1984 [12] or the ASAS classification criteria of 2009 [13]. All patients were exclusively treated by nonsteroidal anti-inflammatory drugs (NSAIDs), For NSAIDs are the first choice for axial SpA patients, and other drugs, such as sulfasalazine, methotrexate, and biological agents may affect the hematopoietic function of bone marrow and have a greater impact on peripheral blood cell counts. Patients with autoimmune diseases such as Sjogren’s syndrome (SS), systemic lupus erythematosus (SLE), rheumatoid arthritis (RA) and psoriasis, malignant diseases, end-stage kidney diseases, liver diseases, acute myocardial infarction, hypertension, diabetes, cerebrovascular diseases were excluded.

### Sacroiliitis X-ray staging of the axial SpA patients

The stage of sacroiliitis was assessed using X-ray and staged from I to IV as follows: stage I with suspicious sacroiliitis; stage II with vague margin of sacroiliac joint, slightly sclerotic and minimally invasive lesions, and unchanged joint space; stage III with moderate or progressive sacroiliitis, accompanied by one or more following changes, including sclerosis of proximal articular area, narrowing/widening of joint space, bone destruction or partial ankylosis; and stage IV with complete joint fusion or ankylosis with or without sclerosis. According to the sacroiliitis classification, early-stage (Stage I and II) patients were defined as non-radiographic axial SpA group, and late-stage (Stage III and IV) patients were defined as radiographic axial SpA group.

### Healthy controls

Healthy controls (HCs) included 55 males and 23 females with a mean age of 40 (30–53) years. These subjects were selected from individuals who underwent a physical examination at the Physical Examination Center of Taizhou Hospital (Zhejiang, China) and the sex and age of these subjects were matched with axial SpA patients. All subjects were healthy without any disease and were not taking drugs that affect bone metabolism, such as hormone replacement therapy.

### Biological detection and imaging system

Fasting blood samples were obtained from all included subjects, whereas X-ray imaging was acquired simultaneously from axial SpA patients. Blood routine tests were assessed using the Mindray BC6800-plus (China) automatic blood analyzer. ESR was detected using the ALifax Tes1(Italy) automatic blood analyzer. CRP was detected by Immage 800 (Beckman Coulter, USA). ALT, AST, TBIL and Alb/Globin (A/G) were detected using the AU5800 (Beckman Coulter, USA) automatic biochemical analyzer. X-ray images were obtained using the Digital X-ray imaging system (DR) (Philips, Holland).

### Statistical analyses

All statistical analyses were performed using SPSS version 19.0 (SPSS Inc., Chicago, IL), and all graphs were drawn using GraphPad Prism 8. Kolmogorov-smirnov test was used to check the normality of the distribution. Continuous variables that conform to a normal distribution were expressed as the mean ± standard deviation, and t tests were used for comparison between groups. The median (P25-P75) was used for non-normal distributions, and Mann Whitney U test and Kruskal Wallis tests were used for comparisons between groups. Categorical variables were expressed as numbers (percentage), and comparisons between groups were performed using chi-square or Fisher’s exact tests (the theoretical frequency in one or more square is less than 5). Receiver operating characteristic (ROC) curve analysis with calculation of area under curve (AUC) and 95% confidential interval (CI) was used to determine the role of LMR in the diagnosis of axial SpA. Moreover, the optimal cut-off value was calculated using Youden’s index based on specificity and sensitivity. The correlations between LMR and other indicators were assessed using Pearson correlation or Spearman correlation analysis for normal and non-normally distributed datas, respectively. *P* ≤ 0.05 was considered to indicate statistical significance.

## Results

### Baseline characteristics of the included subjects

Seventy-eight axial SpA patients [51 males, 27 females; mean age (range): 41 (29–52) years] and 78 healthy controls [55 males and 23 females with a mean age (range) of 40 (30–53) years] were included in this study. Blood-routine test indexes, ESR and the serum levels of CRP in both groups were assayed, and compared between axial SpA and HCs. Higher NLR, RDW, PLR, MPV, ESR, and CRP and lower RBC, Hb, Hct, LMR, ALT, AST, TBIL and A/G levels were noted in the axial SpA group compared to healthy controls (*P < 0.05*), and the differences were significant. (Table 1).

**Table 1:** Comparison of baseline characteristics in axial SpA patients and healthy controls

Characteristics	Axial SpA (n1 = 78)	Healthy controls (HCs) (n2 = 78)	*P*-value
Age (years)	41 (29–52)	40 (30–53)	0.783
Sex (Male, %)	51 (65.4)	55 (70.5)	0.607
WBC(× 10^9^/L)	7.2 ± 2.3	6.1 ± 1.2	< 0.001
LMR	3.81 ± 1.87	5.98 ± 1.65	< 0.001
NLR	2.63 (1.71–3.55)	1.67 (1.38–2.11)	< 0.001
RBC(× 10^12^/L)	4.21 ± 0.72	5.03 ± 0.42	< 0.001
Hb(g/L)	116 (101–38)	150 (140–159)	< 0.001
Hct	0.361 ± 0.061	0.450 ± 0.038	< 0.001
RDW (%)	13.4 (12.5–14.3)	12.5 (12.2–12.8)	< 0.001
PLR	152.2 (123.2–201.4)	124.6 (102.8–154.6)	< 0.001
MPV (fl)	10.2 (9.6–10.7)	9.5 (8.5–10.5)	0.002
ESR (mm/h)	37 (20–75)	6 (3–9)	< 0.001
CRP (mg/L)	32.1 (14.1–76.0)	2.9 (1.5–4.9)	< 0.001
ALT (U/L)	16.5 (10.0–25.3)	20.5 (16.0–31.3)	0.003
AST (U/L)	18.5 (15.0–25.5)	22.0 (22.0–26.3)	0.001
A/G	1.4 (1.2–1.6)	1.8 (1.7–2.0)	< 0.001
TBIL(μmol/L)	9.34 (6.35–14.23)	15.85 (13.08–19.52)	< 0.001

*WBC* White blood cells; *LMR* Lymphocyte-monocyte ratio; *NLR* Neutrophil-to-lymphocyte ratio; *RBC* Red blood cells; *Hb* Hemoglobin; *Hct* Hematocrit; *RDW* Red cell distribution width; *PLR* Platelet to lymphocyte ratio; *MPV* Mean platelet volume; *ESR* Erythrocyte sedimentation rate; *CRP* C-reactive protein; *ALT* Alanine aminotransferase; *AST* Aspartate Aminotransferase; *A/G* Albumin/globulin ratio; *TBIL* Total bilirubin

### Correlation between LMR and other laboratory parameters in axial SpA patients

**Fig. 1:**
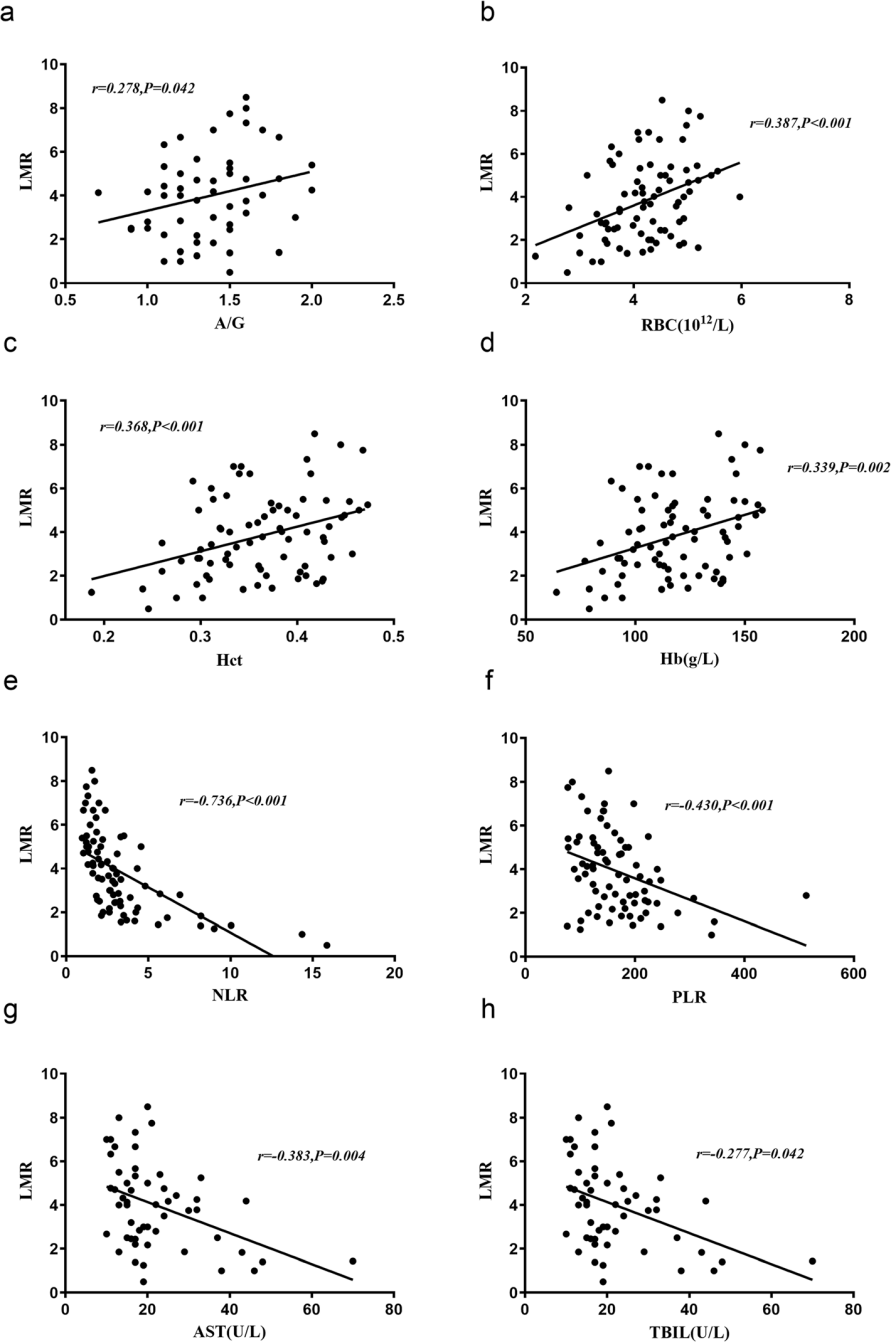
Correlations analyses between LMR and different laboratory parameters including A/G, RBC, Hct, Hb, NLR, PLR, AST and TBIL. Pearson’s correlation was used for LMR, RBC and HCT, and Spearman’s correlation was used for other indexes

We further analyzed the correlation between LMR and other laboratory parameters in axial SpA patients. Positive correlations were observed between LMR and RBC (r = 0.387, *P* < 0.001), Hb (r = 0.339, *P* = 0.002), Hct (r = 0.368, *P* < 0.001) and A/G (r = 0.278, *P* = 0.042), whereas negative correlations were observed between LMR and NLR(r = − 0.736, P < 0.001), PLR (r = − 0.430, P < 0.001), AST (r = − 0.383, *P* = 0.004) and TBIL (r = − 0.277, *P* = 0.042). (Fig. 1).

### ROC curve

**Fig. 2:**
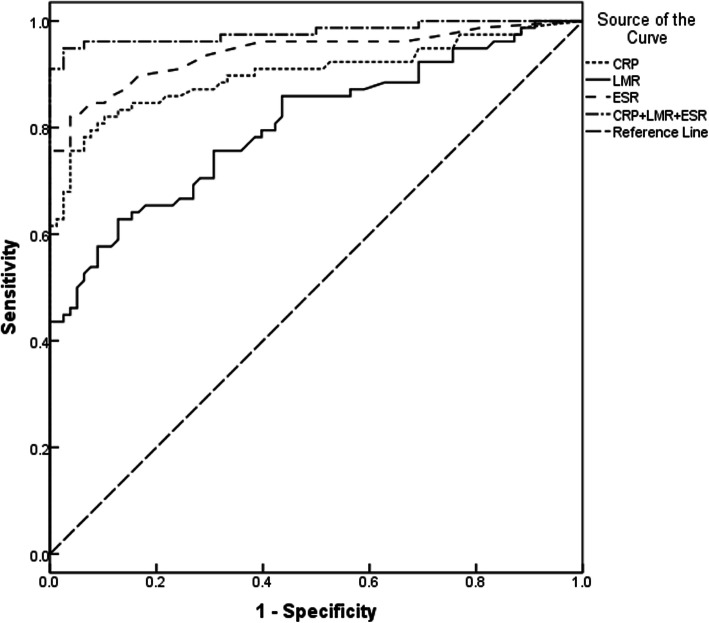
Receiver operating characteristic (ROC) curve analysis of CRP, ESR and LMR in the diagnosis of axial SpA

ROC curves were used to explore the efficiency of clinical indicators in the diagnosis of axial SpA. Indicators with AUC values greater than 0.600 are shown in Table 2. The AUC (95% CI) for LMR was 0.803 (0.734–0.872), and similar values were noted for ESR and CRP [0.937(0.895–0.978), 0.899(0.845–0.954)]. Based on the optimal cutoff values (LMR = 4.26) calculated from the ROC curves, a sensitivity of 62.8% and a specificity of 87.2% was obtained. The AUC (95%CI) for ESR, CRP and LMR in the combined diagnosis of axial SpA were 0.975(0.948–1.000), with the sensitivity and specificity of 94.9 and 97.4%(Fig. 2).

**Table 2:** Comparison of areas under the ROC curve of clinical indicators for diagnosis of axial SpA

	AUC	*P*
ESR (mm/h)	0.937 (0.895–0.978)	*P* < 0.001
CRP (mg/L)	0.899 (0.845–0.954)	*P* < 0.001
LMR	0.803 (0.734–0.872)	*P* < 0.001
RDW (%)	0.773 (0.696–0.849)	*P* < 0.001
NLR	0.718 (0.634–0.801)	*P* < 0.001
PLR	0.687 (0.602–0.772)	*P* < 0.001
A/G	0.648 (0.548–0.747)	0.002
MPV (fl)	0.639 (0.550–0.729)	0.003
ESR + CRP + LMR	0.975 (0.948–1.000)	*P* < 0.001

**Table 3:** Comparison of characteristics between the non-radiographic axial SpA and radiographic axial SpA groups

	Non-radiographic axial SpA (n1 = 43)	Radiographic axial SpA (n2 = 35)	*P*-value
**WBC (**×10^9^/L**)**	**6.5 ± 2.0**	**8.0 ± 2.5**	**0.007**
CRP (mg/L)	31.0 (12.8–69.8)	34.5 (14.7–80.9)	0.786
ESR (mm/h)	40 (18–83)	35 (20–74)	0.637
TBIL(μmol/L)	9.00 (6.10–13.25)	9.80 (6.40–14.85)	0.271
**NLR**	**2.38 (1.62–3.10)**	**3.00 (1.85–5.60)**	**0.046**
PLR	153.1 (128.1–201.1)	164.0 (112.4–217.5)	0.964
**LMR**	**4.21 ± 1.88**	**3.32 ± 1.77**	**0.035**
RBC (×10^12^/L)	4.34 ± 0.65	4.05 ± 0.77	0.078
Hb (g/L)	117.0 (103.0–140.0)	115.0 (94.0–133.0)	0.250
Hct	0.370 ± 0.052	0.351 ± 0.070	0.198
ALT (U/L)	16.0 (8.0–27.0)	17.0 (11.0–21.0)	0.889
AST (U/L)	17.0 (13.0–22.5)	19.0 (15.5–32.5)	0.152
A/G	1.4 **±** 0.3	1.3 **±** 0.3	0.371

*WBC* White blood cells; *LMR* Lymphocyte-monocyte ratio; *NLR* Neutrophil-to-lymphocyte ratio; *RBC* Red blood cells; *Hb* Hemoglobin; *Hct* Hematocrit; *RDW* Red cell distribution width; *PLR* Platelet to lymphocyte ratio; *MPV* Mean platelet volume; *ESR* Erythrocyte sedimentation rate; *CRP* C-reactive protein; *ALT* Alanine aminotransferase; *AST* Aspartate Aminotransferase; *A/G* Albumin/globulin ratio; *TBIL* Total bilirubin

### Comparison of characteristics between the low X-ray stage and high X-ray stage group

A total of 43 and 35 patients were included in the non-radiographic axial SpA group (stage I-II) and radiographic axial SpA group (stage III-IV), respectively. The comparison results revealed higher WBC (8.0 ± 2.5 vs. 6.5 ± 2.0) and NLR [3.00 (1.85–5.60) vs.2.38 (1.62–3.10)] levels and lower LMR levels (3.32 ± 1.77 vs. 4.21 ± 1.88) in the radiographic axial SpA group compared to the non-radiographic axial SpA group (Table 3).

### LMR in different X-ray stages of axial SpA patients

**Fig. 3:**
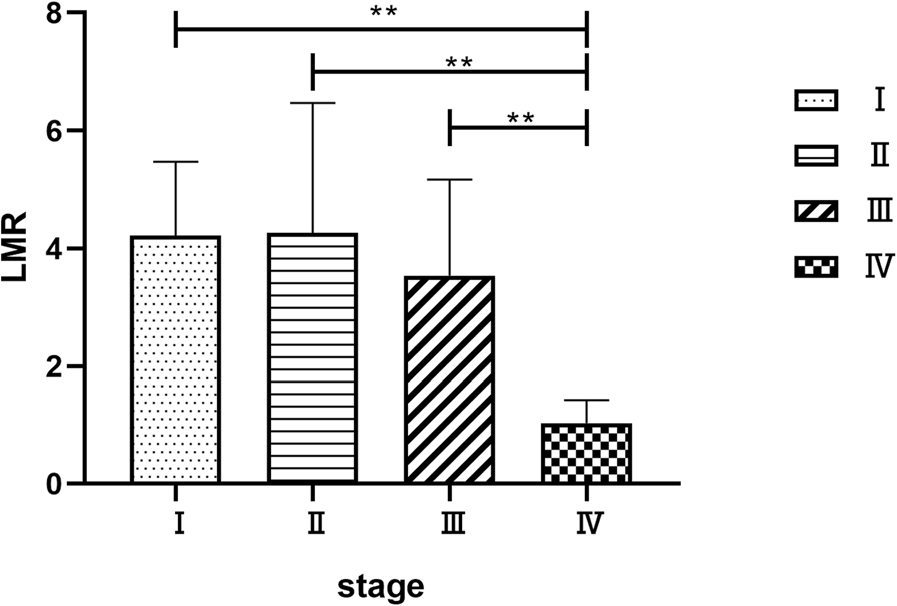
LMR values in axial SpA patients with different X-ray stages. Significantly differences were found between patients with different stages. **: *P* < 0.01

Further staging of axial SpA patients based on X-ray imaging resulted in the allocation of 16, 27, 30 and 5 patients into stages I, II, III and IV, respectively. There were no statistical differences in LMR values among axial SpA patients of stages I, II and III, but LMR values of stage IV patients were lower than other stages (*P* < 0.01). (Fig. 3).

## Discussion

In previous studies, recently developed inflammatory and immunological indicators such as NLR and the platelet-to-lymphocyte ratio (PLR) have been verified as diagnostic makers of disease activity and severity in various disorders. Peng et al. indicated that the combined use of NLR, PLR and CEA could represent a good diagnostic biomarkers for colorectal cancer, and positive correlations were found between the TNM stage and NLR or PLR [14]. In addition, Zhao et al. also observed that NLR was correlated with knee recurrence after arthroscopic surgery combined with local radiotherapy [15]. A recent study of RA patients with and without rheumatoid arthritis-associated interstitial lung disease (RA-ILD) by Chen et al. revealed that PLR and NLR exhibited a statistically significant positive correlation with DAS28 and PLR could be used to diagnose RA and RA-ILD and distinguish RA-ILD patients from RA patients and healthy subjects [16].

In recent years, another indicator LMR has attracted considerable attention in the diagnosis and prognosis of many diseases such as cancers or various immunological diseases. Rajwa et al. found that urothelial bladder cancer patients treated with radical cystectomy with lower LMR values exhibited a greater risk for developing postoperative in-hospital complications [17]. Du et al. showed that the LMR was an inflammatory marker that is effective in disease activity evaluation in patients with RA and RA differentiation from other arthritis condition [18].

The present study revealed a decreased LMR in axial SpA patients compared to healthy controls, especially in axial SpA patients with high X-ray stages. Furthermore, the correlations between LMR and other axial SpA related indicators revealed that LMR was positively correlated with RBC, Hb, Hct and A/G and negatively correlated with NLR, PLR, AST and TBIL.

Anemia is a common phenomenon in the process of chronic inflammation and is also found in axial SpA patients, and the mechanisms were attributed to the inhibitory effects of cytokines secretion. Tumor necrosis factor alpha(TNF-α) could block the effects of Erythropoietin(EPO) on CD34(+) hematopoietic stem/progenitor cells [19]. Increased hemoglobin level were observed in axial SpA patients with significant improvement of physical function and fatigue [20]. Thus, hemoglobin levels could reflect axial SpA activity and severity. Specifically, reduced Hb levels indicate reduced disease severity.

As a major component in serum protein, serum albumin was used to reveal long-standing malnutrition and was also associated with systemic inflammation [21]. Globulin is the carrier of sex hormones, and globulin levels combined with levels of pro-inflammatory proteins (including complement components, immunoglobulin, CRP, interleukin, TNF) are reflective of the inflammatory state [22]. A/G is based on serum albumin and globulin levels and reflects immune nutritional status and systemic inflammatory reactions with more accuracy compared with either indicator alone. A higher A/G value indicates a good malnutrition status and low hormone levels. Besides, Lin et al. also showed that a low A/G level was significantly correlated with high total bilirubin levels but low hemoglobin levels [23]. These results were consistent with the results obtained in this study.

Lymphocytes play an important role in immunology. Although different subsets of T cells are associated with poor tumor prognosis [24, 25], high absolute lymphocyte counts are associated with good prognosis in gastric cancer patients [26]. An increased number of monocytes was associated with poor prognosis in various types of tumors [27, 28].

Monocytes could differentiate into tumor-associated macrophages (TAMs) in the tumor microenvironment [29]. TAMs could promote tumor angiogenesis and tumor growth by secreting TNF-α [30]. Therefore, LMR may be associated with a good axial SpA prognosis because a higher of LMR could result in reduced inhibitory effects of TNF-α on EPO secretion as well as higher hemoglobin levels and A/G ratios. In contrast, lower NLR, PLR, total bilirubin levels and direct bilirubin levels were also observed, supporting the results of this study.

Liver toxic effects could result from systemic rheumatic diseases and therapeutic drugs. Hepatic involvement is a severe type of extra-articular manifestations in various rheumatic diseases. Hepatotoxicity is commonly observed with the use of NSAIDs and disease-modified anti-rheumatic drugs (DMARD) as immunosuppressants [31]. In our study, we only included axial SpA patients who were treated by NSAIDs; Therefore, the negative correlation between LMR and AST was reasonable.

Regarding the correlations we observed between axial SpA and LMR, we further discussed the diagnostic value of LMR in axial SpA prognosis. ROC curve analysis showed that LMR had a high diagnostic value for axial SpA second only to ESR and CRP. The combined diagnostic AUC of LMR, ESR and CRP for axial SpA was 0.975 with a sensitivity and specificity of 94.9 and 97.4%, respectively. Based on X-ray staging, axial SpA patients were divided into the non-radiographic axial SpA and radiographic axial SpA groups. WBC and NLR levels were higher in the radiographic axial SpA groups, whereas LMR levels were lower. Based on these observations, it can be inferred that LMR is associated with the X-ray stage of sacroiliitis in axial SpA patients.

The relationship between LMR and X-ray staging in axial SpA is rarely reported in previous publications. In our research, we classified axial SpA patients as stage I to IV according to X-ray imaging to discuss the associations between LMR and the severity of sacroiliitis. The value of LMR decreased as X-ray staging increased, indicating the role of LMR in determining axial SpA severity.

The main limitation of our study was it only assessed patients from a single center. Therefore, multicenter prospective study is needed for further verification of our results. Secondly, we could not obtain scoring criteria related to axial SpA activity such as BASDAI and ASDAS activity indexes, given the lack of or incomplete clinical datas of axial SpA patients. Third, the sample size was relatively small given the low prevalence and we only included axial SpA patient treated by NSAIDs in the present study. Fourth, most of axial SpA patients included in the study exhibited a lower stage of activity and disease severity, and more sensitive imaging techniques are needed.

## Conclusion

In conclusions, we found that LMR is a rapid, cheap and nonradioactive parameter that can be used to auxiliary diagnosis axial SpA. Furthermore, LMR may represent an important tool for the assessment of disease activity and X-ray stage of sacroiliitis in axial SpA patients. However, prospective studies on large sample sizes are needed for further validation.

## Data Availability

The datasets used and/or analyzed during the current study are available from the corresponding author on reasonable request.

## Abbreviations

Axial SpA: Axial Spondyloarthritis
HCs: Healthy controls
NLR: Neutrophil lymphocyte ratio
RDW: Red blood cell distribution width
PLR: Platelet lymphocyte ratio
MPV: Mean platelet volume
ESR: Erythrocyte sedimentation rate
CRP: C-reactive protein
RBC: Red blood cell
Hb: Hemoglobin
Hct: Hematocrit
LMR: Lymphocyte-monocyte ratio
ALT: Alanine aminotransferase
AST: Aspartate aminotransferase
TBIL: Total bilirubin
A/G: Albumin/globulin
AUC: Area under curve
CI: Confidential interval
TAM: Tumor associated macrophages
TNF-α: Tumor necrosis factor alpha
EPO: Erythropoietin
NSAIDs: Non-steroidal anti-inflammatory drugs
DMARD: disease-modified anti-rheumatic drugs
RA: Rheumatoid arthritis
